# Impacts of COVID-19 on the Utilization of the Medicare Hospice Benefit

**DOI:** 10.1093/geroni/igab046.241

**Published:** 2021-12-17

**Authors:** Michael Plotzke, Betty Fout, Thomas Christian

**Affiliations:** 1 Abt Associates, Saint Louis, Missouri, United States; 2 Abt Associates, Rockville, Maryland, United States; 3 Abt Associates, Cambridge, Massachusetts, United States

## Abstract

The Coronavirus Disease 2019 (COVID-19) Public Health Emergency (PHE) has had a substantial impact on the provision and utilization of healthcare services. Given the high mortality rate associated with COVID-19 amongst older adults, COVID-19 is likely to have a profound impact on all hospice users due to disruptions in providing services. Our work describes how Medicare beneficiaries have utilized the Medicare Hospice Benefit (MHB) during the PHE and how that compares to utilization of the MHB prior to the PHE. We conducted a retrospective analysis of 100% Part A and Part B Fee-for-Service (FFS) Medicare claims from January 1, 2019 – December 31, 2020. We identified approximately 42.3 million unique Medicare FFS beneficiaries from January 2019 through December 2020. Of these, 1.6 million (3.8%) had at least one hospice claim and 1.7 million (4.0%) had at least one Medicare Part A or Part B claim with a COVID-19 diagnosis during the same time period. The rate of COVID-19 amongst FFS Medicare patients who utilized hospice was 8.3%. Average per-beneficiary per-month hospice visits fell by 28.2% for aides and 15.4% for nurses from December 2019 (7.1 aide visits, 6.5 skilled nursing visits) through December 2020 (5.1 aide visits, 5.5 skilled nursing visits). CMS should continue to monitor the prevalence of COVID-19 amongst hospice users and measures of hospice utilization amongst all hospice users in order to better understand how the PHE impacts the provision of the MHB and ensure beneficiaries continue to have access to needed services.

